# Pesticide, Veterinary Medicines, and Microplastics: Bipartite and Tripartite Interactions Drive the Transformation of Albendazole and Pyraclostrobin in Agricultural Soils

**DOI:** 10.1021/acs.jafc.5c09441

**Published:** 2025-12-18

**Authors:** Eleni R. Lamprou, Hongfei Liu, Myriel Cooper, Stathis Lagos, Joana MacLean, Clemence Thiour-Mauprivez, Aymé Spor, Fabrice Martin-Laurent, Matthias C. Rillig, Dimitrios G. Karpouzas

**Affiliations:** † University of Thessaly, Department of Biochemistry and Biotechnology, Laboratory of Plant and Environmental Biotechnology, Larissa, Viopolis 41500, Greece; ‡ INRAE, Agroecologie -17 Rue Sully, Dijon 21000, France; § Univ. Bourgogne Europe Institut Agro, INRAE, Agroécologie, Dijon 21000, France; ∥ 9166 Freie Universität Berlin, Institute of Biology, Altensteinstraße 6, Berlin 14195, Germany

**Keywords:** albendazole, pyraclostrobin, microplastics, dissipation, LDPE, biodegradable plastics

## Abstract

Soil constitutes a major sink for microplastics (MPs). In agricultural soils, microplastics co-occur with pesticides and veterinary medicines like anthelminthics (AHs). Little is known regarding the influence of microplastics on the dissipation of these organic pollutants. We hypothesized (a) that microplastics due to their hydrophobic surfaces would affect the dissipation of the anthelminthic albendazole (ABZ) and the fungicide pyraclostrobin (PYR), and (b) the outcome of this interaction will vary depending on the type (PBAT-based, Starch-based, and LDPE-based) and the concentration (0.1 and 0.01%) of plastics. (c) Besides microplastics, the co-occurrence of ABZ and PYR will influence each other’s dissipation. We tested the dissipation of ABZ and PYR in the presence and absence of microplastics in three soils. The dissipation of ABZ was accelerated in the presence of microplastics in Greek soil (DT_50_ 2.8–8.2 days vs 13.9 days in the control) but not in the other two soils, while microplastics had no effect on the dissipation of PYR in all three soils. No systematic type- or concentration-driven effect of microplastics on ABZ and PYR soil dissipation was observed in the three soils. Regardless of microplastics’ presence, ABZ delayed PYR dissipation in Greek soil (DT_50_ 47.5 to 99.4 days), an effect further exacerbated in the presence of microplastics (DT_50_ 47.0–59.9 to 72.1–117.5 days). We suggest that complex tripartite interactions between pesticides–anthelminthics–microplastics are operative in agricultural soils affecting the dissipation of pesticides and anthelminthics. These interactions are not considered in the current framework of chemical risk assessment, and they are expected to have serious implications, undermining environmental quality and soil health.

## Graphical abstract

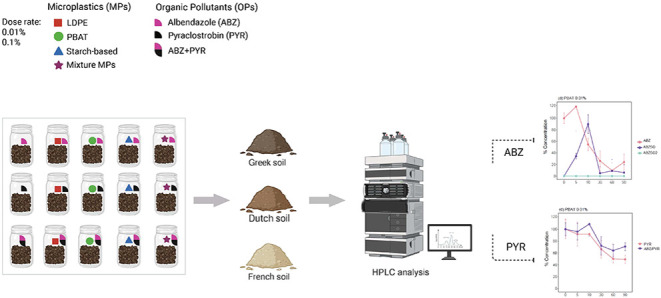

## Introduction

Plastics have become integral to modern life, with their annual production increasing, marking the current era as the “Plastic Age”.^1^ In 1950, global plastic production was approximately 1700 megatons, which increased to over 380 megatons by 2015 and is expected to reach 1800 megatons by 2050.^2^ Simultaneously, about 60% of manufactured plastics are eventually released into the environment as waste, resulting in widespread environmental contamination.^3^ This pervasive plastic pollution represents a significant global threat to ecosystems, wildlife, and human health, fitting the profile of a “poorly reversible pollutant” due to its persistent nature in the environment and the challenge of significantly reducing its emissions.^4^

Plastic waste accumulates in marine^5^ and terrestrial ecosystems.^6^ The former had been the primary focus of early environmental research,^7–9^ whereas interest has shifted to soil ecosystems in recent years and particularly to agricultural soils.^6^ Anthropogenic activity can introduce microplastics to the soil intentionally, through irrigation systems, greenhouse coverings, and the use of plastic mulch, or unintentionally, via manure or compost applications, wastewater, seed, pesticide, and fertilizer coatings.^10,11^ Among these, plastic mulching constitutes the most significant contributor to plastic pollution in agricultural soils.^12^

Mulching films are mainly composed of low-density polyethylene (LDPE).^13^ LDPE is chemically inert, and it is slowly degraded, mainly via abiotic (e.g., UV irradiation) processes.^14,15^ Complete removal of these mulch films from the fields is time-consuming and expensive, and only a few countries in Europe systematically collect and recycle used agricultural plastics. As a consequence, plastic films are left on the field constituting a major environmental threat.^16^ In response to this, biodegradable plastics were introduced in the market.^17^ Such an example is poly­(butylene adipate-*co*-terephthalate) (PBAT), a synthetic aliphatic–aromatic copolyester derived from fossil resources.^18^ Due to its low production costs, lightweight composition, and mechanical durability, PBAT-based plastics are widely utilized in food packaging, agriculture, and textiles.^19^ In agriculture, PBAT-based mulch films are the most used type after LDPE.^20^ A more recent and popular biodegradable type of plastic film is starch based.^21^ These are complex blends of starch with other biodegradable polylactic acid and PBAT that offer several advantages over LDPE, including biodegradability, high availability, and low cost.^22–25^ Starch-based plastics are used in agriculture, packaging, and the manufacturing of hygienic items.^26,27^ While biodegradable plastics were developed as a more sustainable alternative to nonbiodegradable conventional plastics, they do not undergo complete degradation, leading to greater microplastic accumulation and posing an ecological threat^28^ that can significantly affect the productivity of agricultural soils.^29^

In soil, plastics undergo weathering, giving rise to microplastics,^11^ fragments of less than 5 mm,^30^ that impose direct and indirect adverse effects on the soil biota.^22–25^ Microplastics interact with other organic pollutants that are intentionally (e.g., pesticides) or unintentionally (e.g., veterinary medicines) released in agricultural soils.^31,32^ Most studies have focused on the adsorption of pesticides and antibiotics on plastics. They showed that lipophilic substances are adsorbed onto the hydrophobic surfaces of microplastics, while plastic aging and plastic type play a role in their adsorption affinity.^33^ Much less is known about the effects of microplastics on the soil dissipation of pesticides^34,35^ and antibiotics,^36^ while nothing is known about the interactions of microplastics with anthelminthics, another group of veterinary medicines is frequently detected in agricultural soils^37^ and is known to impose undesirable effects on the soil biota.^38^ All these studies have explored bipartite interactions (i.e., microplastics + pesticide or microplastics + antibiotic),^34–36,39^ while tripartite or more complex pollutant co-occurrence scenarios, which constitute the rule rather than the exception in agricultural soils, have been overlooked.

Regarding anthelminthics, albendazole (ABZ), one of the most frequently used compounds in livestock farming for the control of gastrointestinal nematodes, was considered in our study.^40^ ABZ belongs to the chemical group of benzimidazoles and acts as an inhibitor of mitosis.^41^ Upon its administration, ABZ is excreted by animals either intact or in the form of active transformation products, via feces and urine.^42^ These are stock pilled to be ultimately used as manure introducing ABZ in agricultural soils.^43^ Recent studies have demonstrated the potential of ABZ, once in the soil, to enter and move up in the food chain^44^ and to exhibit adverse effects on soil fauna^45^ and soil microbiota.^46,47^ Regarding pesticides, pyraclostrobin (PYR), a multipurpose strobilurin fungicide accounting for 25% of the global fungicide market,^48^ was used in our study. It acts by binding to cytochrome *b* inhibiting mitochondrial respiration.^49^ Its regular application has resulted in its widespread occurrence in agricultural soils^50^ and estuaries,^51^ while there are reports about its adverse effects on the soil microbiota.^52^

We aimed to investigate the effect of different types of microplastics, conventional, nonbiodegradable, and biodegradable, on the soil dissipation of ABZ and PYR in bipartite and tripartite co-occurrence scenarios. Specifically, we tested the hypotheses that (a) the presence of microplastics may significantly affect the soil dissipation of ABZ and PYR, (b) the effects of microplastics on the soil dissipation of ABZ and PYR may vary among the different types and concentrations of microplastics used, and (c) besides microplastics, the co-occurrence of ABZ and PYR may influence each other’s dissipation rates in soil. To test these hypotheses, we determined the dissipation kinetics of ABZ and PYR in the presence or absence of three types of plastics (LDPE, PBAT-based, and starch-based) and their combination applied at two dose rates in agricultural soil (Greek soil). We extended our study, using the same experimental plan, in two other agricultural soils (Dutch and French soil), where the dissipation of ABZ and PYR was monitored only at selected time points postapplication.

## Materials and Methods

### Pesticides and Anthelminthics

Analytical standards of ABZ (98% purity) and albendazole sulfoxide (ABZSO 98% purity) were obtained from Tokyo Chemical Industry (Zwijndrecht, Belgium). Albendazole sulfone (ABZSO_2_ 97% purity) was purchased from Santa Cruz Biotech (Heidelberg, Germany). Analytical standard PYR (99.9% purity) was purchased from HPC Standards GmbH (Borsdorf, Germany). Stock standard solutions of ABZ, ABZSO, and ABZSO_2_ (mixture of all three) and PYR (1000 mg L^–1^) were dissolved in acetonitrile and used for analytical purposes. Working solutions were prepared by serially diluting the stock solution to obtain concentrations of 20, 10, 5, 2, 1, 0.5, 0.2, 0.1, 0.05, and 0.01 mg L^–1^ for each analyte.

### Soils

Three soils were used in the study. They were selected among a range of other agricultural soils around Europe based on their negligible background contamination levels with microplastics. Specifically, the soils used were analyzed for microplastic detection^53,54^ and showed that none contained particles larger than 50 μm. The first was a sandy clay loam soil collected from an olive orchard in Voukolies village on the island of Crete, Greece, an agricultural area in the Mediterranean region that is a hotspot of climate change. The second was a sandy loam soil obtained from an organic farm in Wageningen, The Netherlands, and the third soil was a clay loam soil collected from a field at the INRAE experimental farm located in Bretenière, eastern France. Details on the main physicochemical properties of the soils used are presented in Table 1. In all cases, topsoil samples (0–10 cm layer) were collected from each sampling site at five selected points using a W-shaped nonsystematic pattern, following the ISO 10381-1 and ISO 10381-2 guidelines.^55^ Immediately after collection, the soil was stored in refrigerators at 4 °C and transported to the laboratory in paper boxes to avoid any cross-contamination with microplastics. Moisture content was measured by oven-drying subsamples at 105 °C for 24 h. Water-holding capacity was assessed gravimetrically by saturating a 20-g soil sample with distilled water in a funnel lined with filter paper, followed by a 24 h drainage.

**Table 1:** Physicochemical Properties of the Soils Studied

soil properties	Greek soil	Dutch soil	French soil
clay (%)	28	33.3	4.6
silt (%)	24	38.7	42.7
sand (%)	48	27	52.7
soil type	sandy-loam	clay-loam	sandy-loam
organic matter content (%)	5.6	4.7	4.0
pH	6.37	8.02	5.9

### Plastics

Three different types of plastics were used in our study: (a) LDPE based, (b) PBAT based, and (c) starch based. In all cases, commercial mulching plastic films were used that did not undergo any aging. The LDPE-based plastic was a mulching black film, 30 μm thick, which was provided by Oerlemans (The Netherlands). The PBAT-based plastic was a mulching black film, 15 μm thick that was enriched in PBAT and contained PLA and other polyesters, and it is characterized as biodegradable (provided also by Oerlemans, Netherlands). The starch-based plastic was a 15 μm-thick black mulching film, which was provided by Novamont (Italy).

Plastic sheets were cryomilled and sieved as described in McColley et al.^56^ The plastic fragments obtained were analyzed using a Mastersizer 3000 from Malvern Instruments Ltd., equipped with a dry powder dispersion unit. The software utilized for the analysis was Mastersizer v3.81. Particles smaller than 250 μm were used in our study. The density of the different plastic types used was 0.93, 1.45, and 1.28 g cm^–3^ for the LDPE based, PBAT based, and starch based, respectively.^57^

### Experimental Setup

Fresh soil was partially air-dried and passed through a 2 mm mesh sieve. Microplastics were introduced into the soil at two different concentration levels, 0.01 and 0.1% w/w (dry weight of soil), which are within the concentration gradient proposed by^58^ to study the ecological effects of plastics on soil biota (0.005–1%). Specifically, the lower concentration is close to the levels recovered in agricultural soils, i.e., 55 ^58,59^ and 63.04 mg kg^–1^ ^60,60^ or calculated based on the application rates of the mulching plastic film, i.e., 0.004%.^61^ The higher concentration was used as a projection of the concentrations of plastics expected to accumulate in soils in the near future.^62^ In both cases, the microplastics were thoroughly mixed in soil by hand and allowed to equilibrate for 3 days. The microplastics were previously washed using 70% ethanol and then exposed to UV light for 30 min to minimize microbial contamination.

Soils were subsequently treated with 5.6 and 4.3 mL of methanol solutions of ABZ (150 mg L^–1^) and PYR (115 mg L^–1^) to achieve a soil concentration of 1 mg kg^–1^. Each of the three soils was amended individually with ABZ (bipartite), PYR (bipartite), and a combination of the two compounds (tripartite). Following the application of these compounds, the soil was left for 1 h to allow for the solvent to evaporate, and water was added to adjust the soil moisture content to 40% of the water-holding capacity. The samples were thoroughly mixed by hand to ensure the homogeneous distribution of the compounds and water. The moisture of the soil was maintained at this level throughout the study, with weekly additions of deionized water being needed. Each soil treatment was placed into sterilized, aerated glass jars, which were incubated in the dark at 20 °C for 90 days. Immediately before the placement of the soil samples in the incubator (day 0), and at regular days thereafter (5, 10, 15, 30, 60, and 90 days), five replicates per treatment were removed and analyzed for ABZ and PYR residues. The time-series analysis of the dissipation of the two compounds was performed in Greek soil, while in the other two soils, the residues of ABZ and PYR were measured only at 30 and 90 days postapplication to verify if our observations are valid across soils.

### Determination of the Residues of ABZ and PYR in Soil

#### Extraction of Compounds from Soil

The residues of ABZ and its transformation products ABZSO and ABZSO_2_ were extracted from soil as described by Lagos et al.^63^ Briefly, 5 g of soil was mixed with 10 mL of acetonitrile and shaken for 1 h on a horizontal platform at 220 rpm. The extract was centrifuged for 5 min at 7500 rpm. The supernatant was collected, and the soil was re-extracted with an additional 10 mL of acetonitrile. The supernatants were combined, filtered through a 0.45 μm hydrophobic PTFE syringe filter, and analyzed by HPLC. PYR residues were extracted from 5 g of soil by using 7 mL of acetonitrile. The extraction process involved two cycles, as described above for ABZ.

### HPLC Analysis

Analyses of the samples from soils A and B for ABZ and of the Greek soil for PYR residues were performed in a Shimadzu HPLC-DAD system equipped with a Grace Smart RP C18 column (150 × 4.6 mm). ABZ was detected at 205 nm, while ABZSO and ABZSO_2_ were detected at 220 nm. The mobile phase consisted of a 20/80 (v/v) mixture of acetonitrile and a 0.1% phosphoric acid solution, with a flow rate of 1 mL min^–1^. Under these conditions, the retention times were 27.5 min for ABZ, 10.1 min for ABZSO_2_, and 4.5 min for ABZSO. PYR was detected at 275 nm, and the mobile phase consisted of a mixture of 80/20 v/v acetonitrile and 0.1% phosphoric acid solution, with a flow rate of 1 mL min^–1^ as well. The retention time of the fungicide was 4.2 min.

Analyses of ABZ residues in French soil and of PYR residues in French and Dutch soil were performed in an Agilent 1100 HPLC system (Agilent Technologies, Courtaboeuf, France) equipped with a reverse-phase column (C18 Zorbax Eclipse Plus, 75 × 4.6 mm, 3.5 μm). ABZ and its transformation products were detected at the same nanometers using the same mobile phase as that described above. The studied compounds were eluted at 28.7 min for ABZ, 13.7 min for ABZSO_2_, and 6.3 min for ABZSO. Regarding PYR, it was detected at 275 nm. The mobile phase consisted of a mixture of 70/20/10 v/v acetonitrile, 0.1% phosphoric acid solution, and methanol at a flow rate of 1 mL min^–1^. With this protocol, PYR had a retention time of 6.4 min.

### Validation of the Analytical Methods

The extraction efficiency was assessed using soil samples fortified with ABZ and PYR at three concentration levels: 5, 1, and 0.05 mg kg^–1^. Each compound X concentration level combination was processed in triplicate. The mean percentage recoveries were 91.7% for ABZ, 91.4% for ABZSO, and 105.2% for ABZSO_2_. The limits of detection (LOD) and quantification (LOQ) were 0.01 and 0.25 mg kg^–1^, respectively, for ABZ, ABZSO, and ABZSO_2_. Similarly, for PYR, the mean percentage recoveries were 90% and the LOD and LOQ were 0.01 and 0.1 mg kg^–1^, respectively.

### Dissipation Kinetics

The dissipation kinetics of ABZ, ABZSO, ABZSO_2_, and PYR in Greek soil were determined using four models as proposed by the FOCUS working group on pesticide degradation kinetics.^64^ These models included the single first-order (SFO) kinetic model and three biphasic models: the hockey-stick (HS) model, the first-order multicompartment (FOMC) model, and the double first-order in parallel (DFOP) model. Biphasic models were used in cases where the SFO kinetic model did not provide an adequate fit to the dissipation data based on the χ^2^ test value (<15%), visual inspection, and the distribution of residuals. Kinetic analysis was conducted using R (R Core Team, 2022) in RStudio version 3.5.0, with the mkin package^65^ version 0.9.47.1. Details on the theory and the equations of the kinetic models used are given elsewhere.^66^

### Statistical Analysis

Residual levels of ABZ and PYR in French and Dutch soils were first tested for fit to ANOVA assumptions (normality of residuals and homogeneity of variances) and then subjected to univariate analysis. On the other hand, differences among treatments in Greek soil were evaluated through comparisons between degradation rate constants (*k*deg or *k*_1_), while DT_50_ was treated as a derived descriptive metric (DT_50_ = ln(2)/*k*). For each treatment, standard deviations (SD) of *k* were reconstructed from the 95% confidence intervals (CI) of the kinetic fits using SE = (Upper–Lower)/(2·*t*_0.975_,*df*) [≈(Upper–Lower)/3.92 for large *df*] and SD = SE·√*N*, where *N* = 3 is the number of independent replicate microcosms per treatment and 3.92 = 2 × 1.96 (95% normal CI width in SE units). Control-versus-treatment comparisons were then performed on *k* using Dunnett-type contrasts implemented via Wald tests with Holm’s adjustment (two-sided, α = 0.05). Regarding the other two soils (French and Dutch), differences in the residual levels of ABZ and PYR between the different treatments at each time point were determined via two-way ANOVA followed by posthoc Tukey’s test (*p* < 0.05). All statistical analyses were conducted using RStudio version 3.5.0 (R Core Team, 2018).

## Results

### Dissipation of ABZ in Soil

#### Greek Soil

The dissipation of ABZ in most cases was described adequately by the SFO kinetic model. Biphasic models like the HS (primarily) and DFOP provided the best fit to the measured data in cases where the SFO model failed to provide an adequate fit (Table 2, Supporting Information Figures S1 and S2). The dissipation of ABZ in all treatments was accompanied by the formation of ABZSO, while low levels of ABZSO_2_ were also detected (Figures 1 and 2). Considering that ABZSO also carries anthelminthic activity, we also calculated the DT_50_ of the total residues of ABZ, which, in this and earlier studies,^37^ are defined as the sum of ABZ + ABZSO + ABZSO_2_.

**Table 2:** DT_50_ Values of Albendazole (ABZ) and of the Sum of Albendazole and Its Two Transformation Products Albendazole Sulfoxide (ABZSO) and Albendazole Sulfone (ABZSO_2_) (Total Metabolites) in the Presence or Absence of Pyraclostrobin (PYR) in Greek Soil Treated with Two Concentration Levels (0.01 and 0.1%) of a Range of Different Microplastics (No MPs, LDPE based, PBAT based, and Starch based) and Their Mixture^a^

albendazole application								albendazole & pyraclostrobin application							
treatment	compound	DT_50_ (days)	kinetic model	X^2^ (%)	*k*_1_ ^b^ (d^–1^)	*k*_2_ ^c^ (d^–1^)	*t*_b_ ^d^ (d^–1^)	treatment	compound	DT_50_ (days)	kinetic model	X^2^ (%)	*k*_1_ ^b^ (d^–1^)	*k*_2_ ^c^ (d^–1^)	*g* ^e^/*t*_b_ ^d^ (d)
no MPs	ABZ	13.9	SFO	10.81	0.05			no MPs	ABZ	7.9	HS	14.3	0.08	0.002	22.39
	total metabolites	14.3	SFO	9.0	0.04				total metabolites	76.4	SFO	11	0.09		
LDPE-based 0.01	ABZ	6.0	SFO	7.3	0.11			LDPE-based 0.01	ABZ	6.4	HS	11.5	0.1	5.431e-12	0.18
	total metabolites	5.9	SFO	6.7	0.11				total metabolites	65.2	SFO	10.4	0.01		
LDPE-based 0.01	ABZ	5.5	SFO	8.4	0.12			LDPE-based 0.01	ABZ	10.1	SFO	13.87	0.06		
	total metabolites	5.5	SFO	8.4	0.12				total metabolites	46.8	SFO	9.2	0.01		
PBAT-based 0.01	ABZ	5.6	SFO	8.9	0.12			PBAT-based 0.01	ABZ	13.2	HS	10.38	0.05	0.008	21.55
	total metabolites	5.5	SFO	8.4	0.12				total metabolites	50.1	SFO	13.9	0.009		
PBAT-based 0.01	ABZ	2.8	SFO	8.0	0.24			PBAT-based 0.01	ABZ	10.5	HS	12.9	0.06	0.006	19.68
	total metabolites	ND							total metabolites	76.4	SFO	11 0	0.09		
starch-based 0.01	ABZ	7.8	HS	12.0	0.08	0.02	10.00	starch-based 0.01	ABZ	6.2	DFOP	12.0	1.430e-01	1.943-01	8.497e-01
	total metabolites	8.8	HS	12.0	0.07	0.02	10.00		total metabolites	85	SFO	7.9	0.008		
starch-based 0.1	ABZ	8.2	HS	13.1	0.07	0.02	10.00	starch-based 0.1	ABZ	6.5	DFOP	12.4	1.724e-01	3.932e-12	7.386e-01
	total metabolites	7.6	HS	10.0	0.09	0.02	10.00		total metabolites	82.7	SFO	14.2	0.08		
MPmixture 0.01	ABZ	6.0	HS	10.7	0.11	0.02	8.94	MPmixture 0.01	ABZ	6.0	HS	7.6	0.11	0.02	8.14
	total metabolites	6.0	HS	10.8	0.11	0.03	6.52		total metabolites	87.8	SFO	8.6	0.07		
MPmixture 0.1	ABZ	3.6	HS	11.8	0.19	0.01	6.54	MPmixture 0.1	ABZ	6.1	HS	3.1	0.13	0.02	7.22
	total metabolites	3.6	HS	11.7	0.19	0.01	6.48		total metabolites	77.4	SFO	10.4	0.08		

a The DT_50_ values were calculated based on the single first-order (SFO) kinetic model and the biphasic models like the hockey-stick (HS) and the double first-order in parallel (DFOP). The chi-square value (χ^2^), a statistical measure of the fit of the model to the observed values, along with the different kinetic parameters per model, is presented.

b *k*_1_: The first-order degradation rate constant (*d* ^–1^) for the SFO model; the rate constant describing the initial phase of the degradation of a chemical according to the biphasic HS model (*k*_1_ = rate constant until *t* = *t*_b_); the rate constant in compartment 1 of the model DFOP.

c *k*_2_: The rate constant (*d* ^–1^) describing the second phase of the degradation of a chemical, which is described by the biphasic HS model (*k*_2_ = rate constant from *t* = *t*_b_); the rate constant (*d* ^–1^) in compartment 2 of the model DFOP.

d *t*_b_: breakpoint (time in days at which rate constant changes for chemicals whose degradation is described by the HS model).

e *g*: it is a dimensionless shape parameter in the FOMC model that describes the degradation rate decreasing over time.

**Figure 1:**
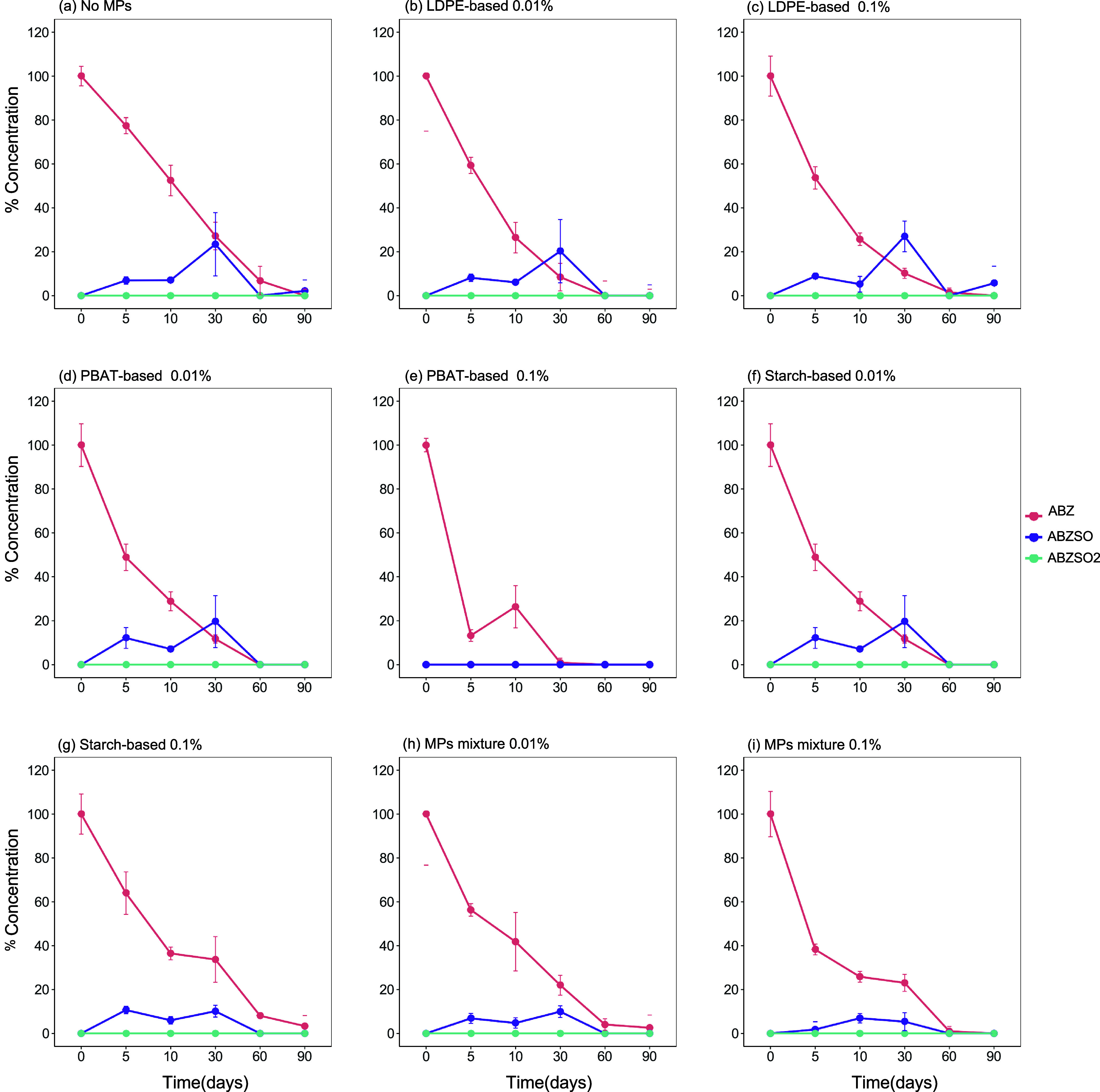
Effects of different types of microplastics (MPs) and their mixture, applied at two different concentrations (0.01 and 0.1%), on the dissipation of Albendazole (ABZ) and its transformation products albendazole sulfoxide (ABZSO) and albendazole sulfone (ABZSO2) in Greek soil: (a) No MPs, (b) LDPE-based 0.01%, (c) LDPE-based 0.1%, (d) PBAT-based 0.01%, (e) PBAT-based 0.1%, (f) Starch-based 0.01%, (g) Starch-based 0.1%, (h) MPs mixture 0.01%, and (i) MPs mixture 0.1%.

**Figure 2:**
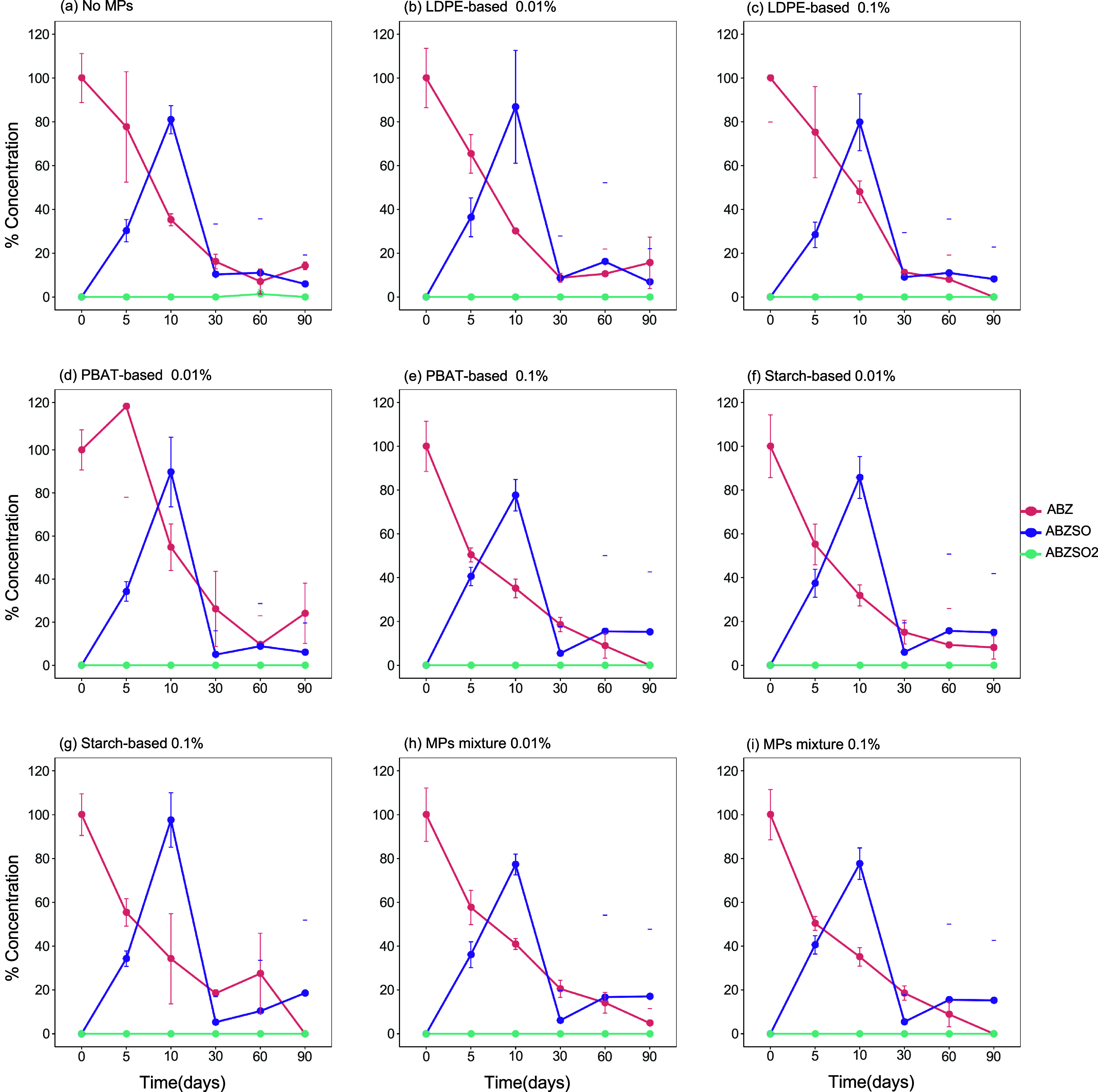
Combined effects of different microplastics (MPs), applied at two concentrations (0.01% and 0.1%), and pyraclostrobin (PYR) on the dissipation of Albendazole (ABZ) and its transformation products albendazole sulfoxide (ABZSO) and albendazole sulfone (ABZSO2) in Greek soil: (a) No MPs, (b) LDPE-based 0.01%, (c) LDPE-based 0.1%, (d) PBAT-based 0.01%, (e) PBAT-based 0.1%, (f) Starch-based 0.01%, (g) Starch-based 0.1%, (h) MPs mixture 0.01%, and (i) MPs mixture 0.1%.

When ABZ was applied alone (without PYR), we noted an acceleration in its dissipation in the presence of microplastics regardless of the plastic type and plastic concentration (Figure 1). This was reflected in the DT_50_ values of ABZ, which varied from 13.9 days in the absence of microplastics (control) to 2.8–8.2 days in the samples treated with microplastics (Table 2). A similar pattern was observed when ABZSO and ABZSO_2_ residues were considered in the calculation of DT_50_. In the absence of microplastics, a DT_50_ value of 14.3 days was obtained compared to the microplastics-treated samples, where DT_50_ values ranged from 3.6 to 8.8 days, except for the PBAT-based (0.1%) treated soils, where no formation of ABZSO and ABZSO_2_ was observed (Table 2).

This enhancement in the dissipation of ABZ was not evident when ABZ was coapplied with PYR, where no significant differences (*p* > 0.05, Wald–Dunnett; Holm-adj.) in the DT_50_ values of ABZ were observed across the different treatments (Figure 2). In particular, the DT_50_ value of ABZ in the control treatment was 7.9 days compared to the microplastic-treated samples, where DT_50_ values of ABZ ranged from 6.1 to 13.2 days (Table 2).

When comparing the DT_50_ values of ABZ applied alone or as a mixture with PYR across the corresponding samples, we did not note an effect (*p* > 0.05, Wald–Dunnett; Holm-adj.) on ABZ dissipation. This was not the case for the total residues of ABZ, where the presence of PYR resulted in a significant increase (*p* < 0.05, Wald–Dunnett; Holm-adj.) in the DT_50_ values in the microplastic-untreated samples (14.3 days for ABZ vs 76.4 days for ABZ + PYR). This effect was more prominent in the microplastic-treated samples, where the DT_50_ values varied from 46.8 to 87.8 days when ABZ was applied with PYR compared to 3.6–8.8 days in the samples where ABZ was applied alone (Table 2).

#### Dutch and French Soil

In the two other soils, the dissipation of ABZ was determined only at 30 and 90 days postapplication; hence, no DT_50_ values were derived (Figure 3). In Dutch soil, we noted a significant decrease (*p* < 0.05, two-way ANOVA followed by posthoc Tukey’s test) in the levels of ABZ from 30 to 90 days in the absence (Figure 3A) or the presence (Figure 3B) of PYR. No significant differences (*p* > 0.05, two-way ANOVA followed by posthoc Tukey’s test) in the residues of ABZ between the control and microplastic-treated samples were evident with the sole exception of the samples treated with PBAT-based microplastics (0.1%) where significantly higher ABZ levels (*p* < 0.05, two-way ANOVA followed by posthoc Tukey’s test) were detected compared to the control at 30 days (Figure 3A). The levels of ABZ recovered in the samples treated with ABZ alone compared to ABZ+PYR did not significantly differ (*p* > 0.05, two-way ANOVA followed by posthoc Tukey’s test), regardless of the different treatments employed (microplastics or not), suggesting no significant bipartite or tripartite interactions of PYR and/or microplastics with ABZ that affect its dissipation.

**Figure 3:**
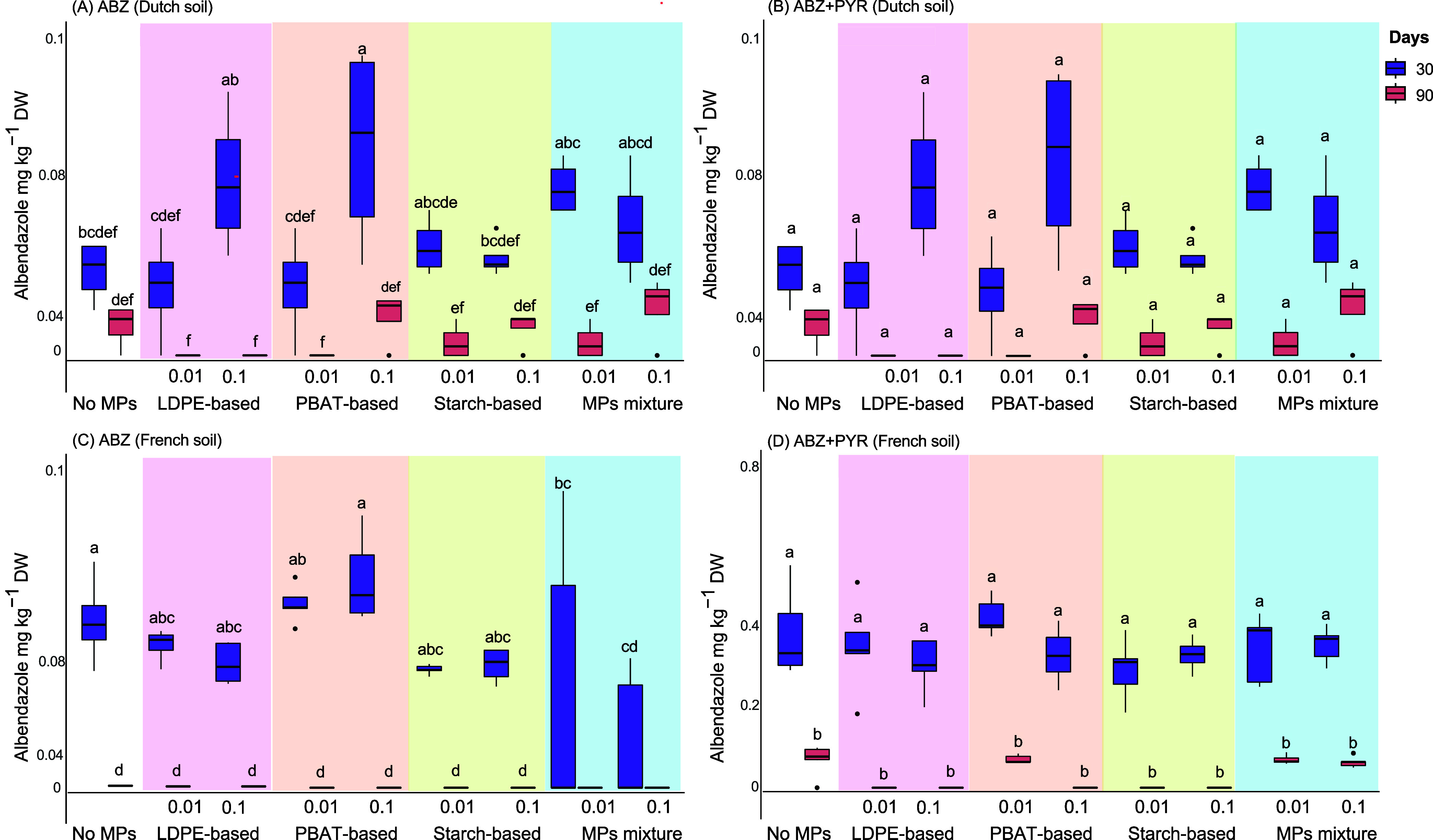
Concentrations of albendazole (ABZ) measured at 30 and 90 days postapplication in the absence (A, C) and presence of pyraclostrobin (PYR) (B, D) in samples from Dutch and French soil that had been treated as follows: (a) No MPs, (b) LDPE-based 0.01%, (c) LDPE-based 0.1%, (d) PBAT-based 0.01%, (e) PBAT-based 0.1%, (f) Starch-based 0.01%, (g) Starch-based 0.1%, (h) MPs mixture 0.01%, and (i) MPs mixture 0.1%. In the boxplot graph, bars designated by the same letters are not significantly different at the 5% level.

In French soil, we noted a complete dissipation of ABZ in all treatments where ABZ was applied alone (Figure 3C) and in most of the ABZ+PYR-treated samples (Figure 3D). When ABZ was applied alone, we did not note a significant effect (*p* > 0.05, two-way ANOVA followed by posthoc Tukey’s test) of microplastics on its dissipation with the sole exception of the samples treated with the mixture of all microplastics where significantly lower concentrations of ABZ (*p* < 0.05, two-way ANOVA followed by posthoc Tukey’s test) were recovered compared to the microplastic-untreated samples (Figure 3C). In the presence of PYR, no significant variations (*p* > 0.05, two-way ANOVA followed by posthoc Tukey’s test) in the residues of ABZ across the different treatments were evident at both sampling days (Figure 3D). It should be noted that regardless of the treatment employed (microplastics or not) significantly higher residual levels of ABZ (*p* < 0.05, two-way ANOVA followed by posthoc Tukey’s test) were present in the samples cotreated with PYR compared to the samples where ABZ was applied alone (Figure 3C,D). This observation implies the establishment of bipartite and possibly tripartite interactions of PYR (and microplastics) with ABZ that result in a slower dissipation of ABZ although the lack of measurements in the respective samples at 0 days does not allow solid conclusions to be reached.

### Dissipation of PYR in Soil

#### Greek Soil

The SFO model provided the best fit to PYR dissipation data across all treatments (Figures 4, S3 and S4). When PYR was applied alone, no significant difference (*p* > 0.05, Wald–Dunnett; Holm-adj.) in its DT_50_ values between the MP-unamended and the microplastic-amended samples was noted with the DT_50_ values varying from 47.5 days in the former to 43.5–59.9 days in the latter (Table 3). A similar pattern was evident when PYR was coapplied with ABZ, where the DT_50_ of PYR was 99.4 days in the microplastic-unamended samples and ranged from 72.1 to 138.1 days in the microplastic-amended samples. The sole exception was the significantly higher DT_50_ of PYR (177.5 days) (*p* < 0.05, Wald–Dunnett; Holm-adj.) in the samples treated with LDPE 0.01%. When comparing the DT_50_ values of PYR in the corresponding samples where the fungicide was applied alone or with ABZ, a significant delay (*p* < 0.05, Wald–Dunnett; Holm-adj.) in the dissipation of PYR was evident in the absence of microplastics (47.5 vs 99.4 days) (Figure 4, Table 3). This deceleration effect was further exacerbated in the presence of microplastics, with the DT_50_ values of PYR ranging from 43.5 to 59.9 days in the microplastic-amended samples treated solely with PYR compared to 72.1–177.5 days observed in the microplastic-amended samples treated with PYR + ABZ (Table 3).

**Table 3:** DT_50_ Values of Pyraclostrobin (PYR) in the Presence or Absence of Albendazole (ABZ) in the Greek Soil That Had Been Treated with Two Concentration Levels (0.01 and 0.1%) of a Range of Different Microplastics (No MPs, LDPE Based, PBAT Based, Starch Based) and Their Mixture^a^

pyraclostrobin application					pyraclostrobin and albendazole application				
treatment	DT50 (days)	kinetic model	X^2^ (%)	*k*_1_ ^b^ (d^–1^)	treatment	DT50 (days)	kinetic model	X^2^ (%)	*k*_1_ ^b^ (d^–1^)
no MPs	47.5	SFO	9.7	0.013	no MPs	99.4	SFO	10.8	0.013
LDPE-based 0.01	47.0	SFO	6.2	0.013	LDPE-based 0.01	177.5	SFO	12.4	0.011
LDPE-based 0.1	53.2	SFO	3.9	0.013	LDPE-based 0.1	72.1	SFO	14.7	0.013
PBAT-based 0.01	57.6	SFO	2.4	0.012	PBAT-based 0.01	109.2	SFO	10.4	0.012
PBAT-based 0.1	43.5	SFO	7.5	0.015	PBAT-based 0.1	138.1	SFO	10.1	0.015
starch-based 0.01	47.9	SFO	6.3	0.014	Starch-based 0.01	102.5	SFO	8.2	0.014
starch-based 0.1	59.9	SFO	5.3	0.011	Starch-based 0.1	91.8	SFO	11.1	0.011
MFs mixture 0.01	49.9	SFO	4.3	0.013	MPs mixture 0.01	89.5	SFO	8.7	0.012
MFs mixture 0.1	50.2	SFO	4.8	0.013	MPs mixture 0.1	83.48	SFO	11.3	0.012

a The DT_50_ values were calculated based on the single first-order (SFO) kinetic model. The chi-square value (χ^2^), a statistical measure of the fit of the model to the observed values.

b *k*_1_: The first-order degradation rate constant (*d* ^–1^) for the SFO model.

**Figure 4:**
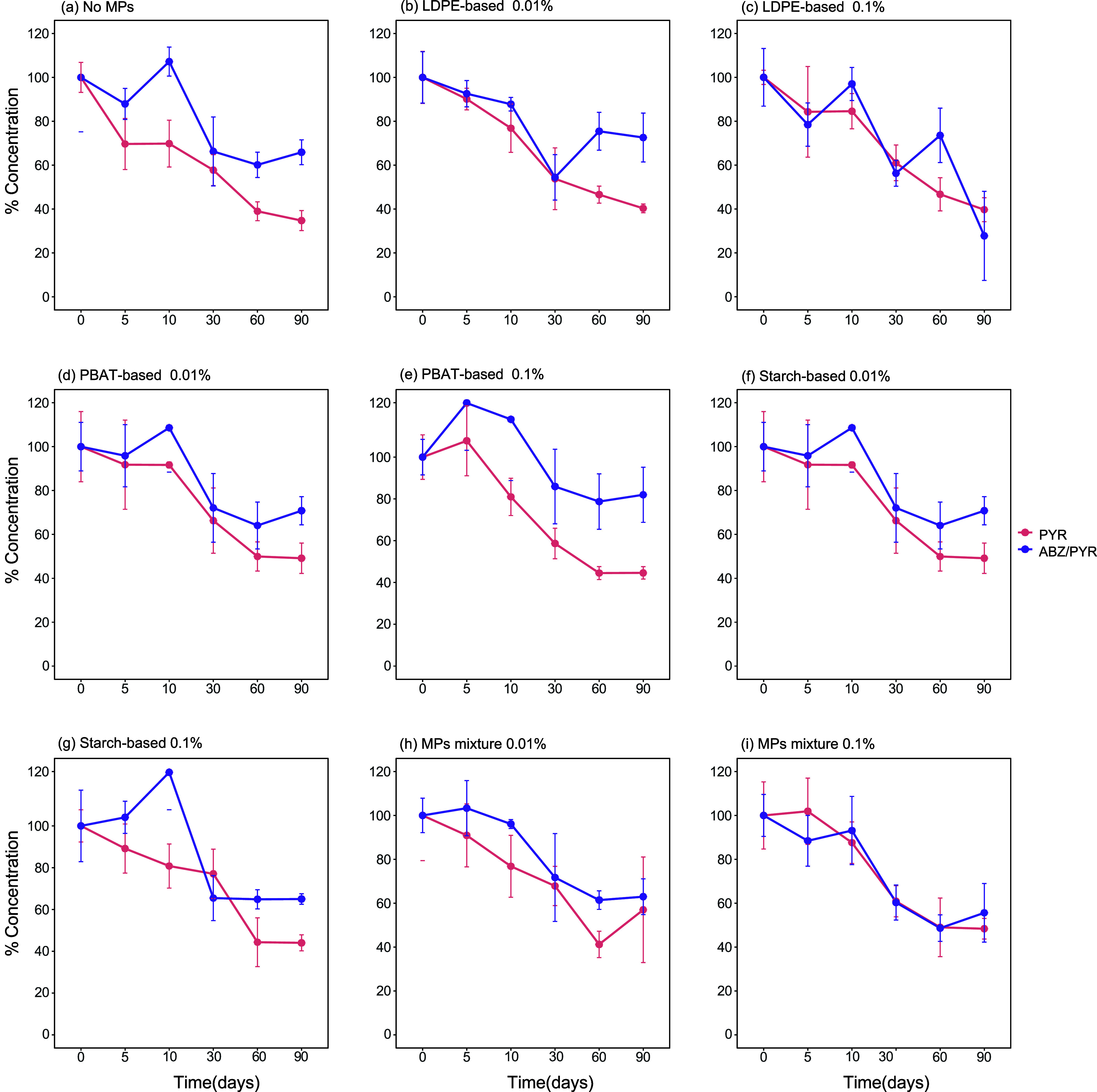
Effects of different types of microplastics (MPs) and their mixture, applied at two different concentrations (0.01% and 0.1%), on the dissipation of pyraclostrobin (PYR), applied either alone or with albendazole (ABZ) in Greek soil: (a) No MPs, (b) LDPE-based 0.01%, (c) LDPE-based 0.1%, (d) PBAT-based 0.01%, (e) PBAT-based 0.1%, (f) Starch-based 0.01%, (g) Starch-based 0.1%, (h) MPs mixture 0.01%, and (i) MPs mixture 0.1%.

#### Dutch and French Soil

In Dutch soil, we noted a significantly higher (*p* < 0.05, two-way ANOVA followed by posthoc Tukey’s test) concentration of PYR in the samples treated with the high dose of LDPE at 30 days, but levels of PYR reverted to concentrations that were equivalent to the rest of the treatments by day 90 (Figure 5A). Apart from this, no significant difference (*p* < 0.05, two-way ANOVA followed by posthoc Tukey’s test) in the levels of PYR across time and treatments was observed, suggesting a slow dissipation of PYR in all treatments from 30 days onward. A similar pattern of nonsignificant difference (*p* > 0.05, two-way ANOVA followed by posthoc Tukey’s test) in the levels of PYR among treatments and time was evident in Dutch soil when PYR was coapplied with ABZ (Figure 5B).

**Figure 5:**
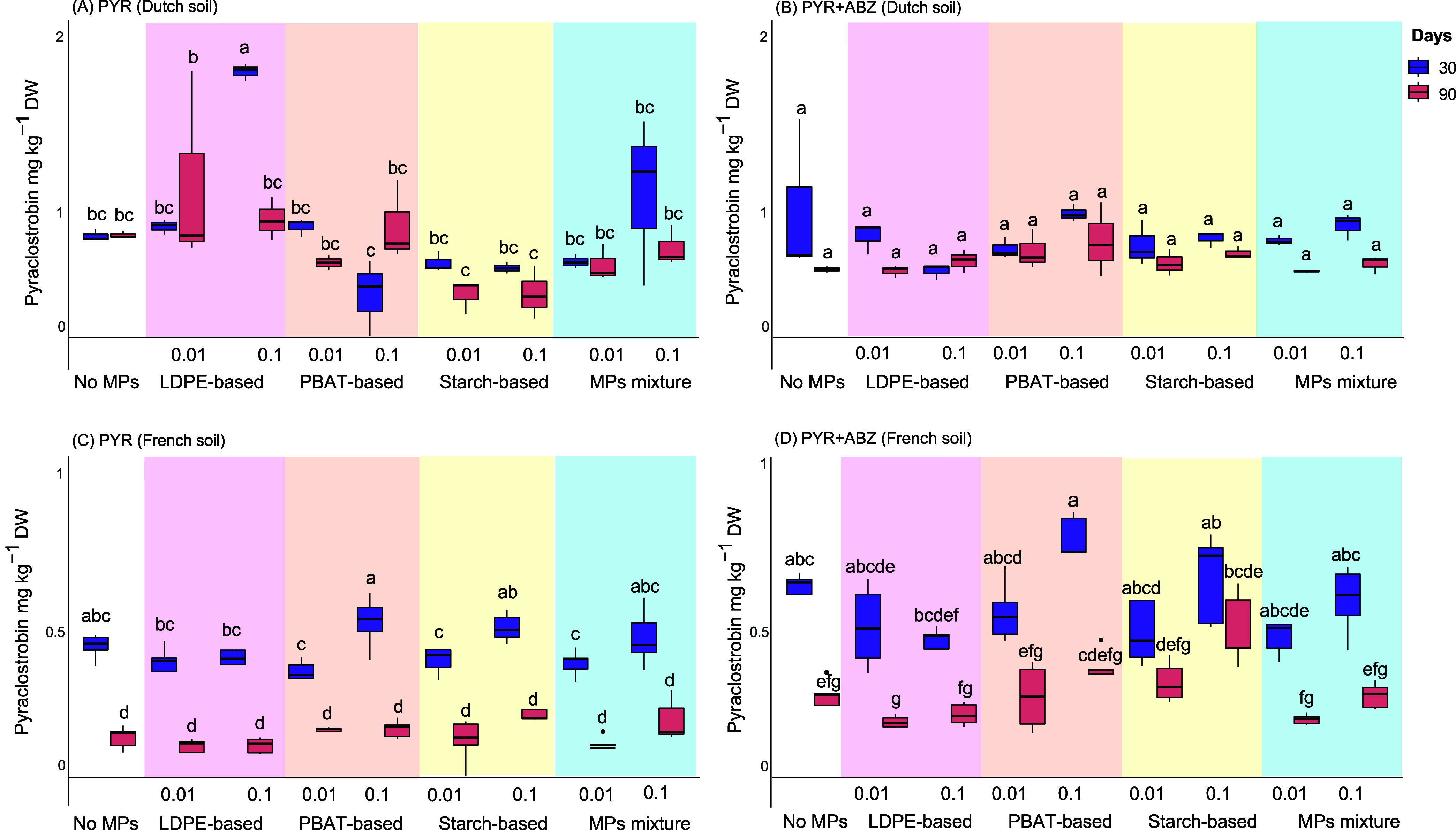
Concentrations of pyraclostrobin (PYR) measured at 30 and 90 days post application in the absence (A, C) and presence of albendazole (ABZ) (B, D) in samples from Dutch and French soil that had been as follows: (a) No MPs, (b) LDPE-based 0.01%, (c) LDPE-based 0.1%, (d) PBAT-based 0.01%, (e) PBAT-based 0.1%, (f) Starch-based 0.01%, (g) Starch-based 0.1%, (h) MPs Mixture 0.01%, (i) MPs mixture 0.1%. In the boxplot graph, bars designated by the same letters are not significantly different at the 5% level.

In French soil, we noted no significant differences (*p* > 0.05, two-way ANOVA followed by posthoc Tukey’s test) in the concentrations of PYR, in the presence or absence of ABZ, across microplastic treatments at 30 and 90 days (Figure 5C,D). On the other hand, we noted a significant reduction (*p* < 0.05, two-way ANOVA followed by posthoc Tukey’s test) in the levels of PYR from 30 to 90 days in all treatments (microplastic-amended and unamended soils) and regardless of the presence of ABZ (Figure 5C,D).

## Discussion

Agricultural soils represent a significant environmental sink for microplastics (microplastics)^67,68^ but also an arena of interactions between microplastics and organic pollutants, like pesticides and antibiotics.^69^ The outcome of these interactions is determined by a range of factors associated with the properties of the interactors and could impede environmental quality and human health.^70,71^ We monitored the effects of a range of microplastics, conventional nonbiodegradable and biodegradable, on the dissipation of ABZ, one of the most used anthelminthics in livestock farming, and PYR, a broad-spectrum fungicide, in three agricultural soils. Our findings showed clear bipartite and tripartite interactions between microplastics and the two organic pollutants that drive the dissipation of ABZ and PYR in a soil-dependent manner, verifying two of our initial hypotheses, whereas the type and the concentration of microplastics did not affect the dissipation of organic pollutants in contrast to our second initial hypothesis.

ABZ and PYR showed largely different soil dissipation rates, regardless of the presence of microplastics. Full-time series dissipation data were obtained only in Greek soil, considered as the most vulnerable to multiple pollutants due to its prior long-term exposure to climate change abiotic stress, as shown recently by^72^, while residual levels of Dutch and French soil at distinct days post application were used to verify our findings across soils. Overall, ABZ did not persist in any of the three soils, with complete dissipation observed in the frame of the incubation in most cases. Its DT_50_ values in Greek soil ranged from 2.8 to 13.9 days, in line with earlier soil studies.^63^ ABZ was transformed to ABZSO and ABZSO_2_, its main TPs as previously observed,^63,73^ although other TPs that might have been formed during the study were not monitored. ABZSO, which also carries anthelminthic activity, was the most abundant transformation product of ABZ being degraded further in soil, while ABZSO_2_ was detected in only trace amounts. On the other hand, PYR was moderately persistent with DT_50_ values in Greek soil ranging from 43.5 to 59.9 days, which are within the range reported in previous studies.^74,75^

Our first hypothesis was that microplastics, acting as a reactive surface to which organic compounds can adsorb^76^ and microorganisms can colonize, may strongly affect the dissipation of ABZ and PYR. In Greek soil, where the dissipation of ABZ was studied in a detailed time-series mode, the presence of microplastics, regardless of type and concentration, significantly accelerated the dissipation of ABZ and its total residues (ABZ + ABZSO + ABZSO_2_). This pattern did not seem to replicate in the other two soils. However, direct comparisons between the three soils should be done with caution since no DT_50_ values are available for the French and Dutch soils. In contrast, the dissipation of PYR was not significantly affected by microplastics presence, regardless of their type and concentration in the three soils.

The distinct behavior of the two compounds in soil in the presence of microplastics could be attributed to their diverse characteristics. ABZ is a nonpersistent compound whose soil dissipation is microbially mediated and positively associated with soil total organic carbon,^63^ while recent studies have indicated its weaker adsorption affinity for plastic surfaces compared to soil colloids.^76^ PYR is a more persistent and less biodegradable compound with high adsorption affinity for soil organic matter and plastic surface sorption,^48,77^ in line with its higher log*K*_ow_ (3.99) compared to ABZ (1.27).^78^ We speculate that the accelerated degradation of ABZ in the presence of microplastics can be attributed to the increased microbial activity facilitated by the microplastics, as they provide a suitable habitat for microorganisms capable of degrading biodegradable compounds,^79^ like ABZ.^63^ Li et al.^80^ showed via meta-analysis that the potential for organic pollutants degradation was remarkably higher in the plastisphere compared to bulk soil, in accord with our hypothesis. Alternatively, the lower adsorption affinity and higher bioavailability of ABZ in microplastic-contaminated soils, recently shown by Tan et al. (2025), might have facilitated the degradation of ABZ by the indigenous microbiota in Greek soil.^81^ This observation was not replicated in the other two soils, whose microbial communities may differ not only in degradation efficiency but also in their functional traits such as the ability to form biofilms, potentially influencing ABZ transformation. Indeed, Lagos et al.^63^ studied the dissipation and transformation of ABZ in a range of different soils, sterilized and nonsterilized, and showed that the degradation potential of soil microbial communities toward ABZ varied significantly among soils.

Regarding the more lipophilic PYR, the lack of any effects on its dissipation might be associated with the low but environmentally relevant levels of microplastics used in our study. In earlier studies, microplastics, when present at levels ranging from 1 to 20% w/w, decelerated the dissipation of simazine^82^ and ciprofloxacin^36^ or altered the dissipation patterns of thiamethoxam.^35^ Conversely, in studies using plastic concentrations equivalent to the ones used in our study, no significant effects on the dissipation of glyphosate and thiacloprid were observed.^83,84^ We suggest that the levels of microplastics used in our study might not offer enough adsorption surfaces to significantly reduce the bioavailability of PYR, which shows higher adsorption affinity on plastic particles over soil,^85^ and hence, its dissipation in all soils is tested.

Our second hypothesis was that the effects of microplastics on the dissipation of ABZ and PYR will vary among the different types and concentrations of microplastics used. Microplastics had a uniform effect on the dissipation of ABZ or PYR, and no consistent motifs of effects among microplastic types and concentrations were observed in the three soils. This was not surprising considering that most studies reporting microplastic-specific and/or dose-dependent effects on the dissipation of pesticides and antibiotics have observed significant alterations at levels much higher than the microplastic levels used in our study (>1%).^34,35,82^

Our third hypothesis was that besides microplastics, bipartite interactions between ABZ and PYR may also have significant effects on their dissipation rates based on earlier studies that have indicated adverse effects of these chemicals on the soil microbiota.^38,46^ Indeed, we identified significant bipartite interactions between the two compounds that affected their soil persistence. Regardless of the presence of microplastics, the dissipation of ABZ in Greek soil was not affected by the coapplication of PYR. A similar pattern was most probably evident in Dutch soil, based on the similar residual levels of ABZ in the presence or absence of PYR. Whereas in French soil, the higher residual levels of ABZ in the samples cotreated with PYR imply a deceleration in the dissipation of ABZ although this should be considered as preliminary evidence in the absence of measurements at 0 days and warrants further verification. However, these observations were supported in Greek soil at the level of ABZ transformation products, where a significant delay particularly in ABZSO dissipation was observed in the presence of PYR. We speculate that the presence of PYR might have adversely affected the soil microbiota, in line with earlier studies,^86–88^ eventually decelerating the transformation of ABZSO, which is primarily microbially mediated.^63^ For example, Hou et al.^52^ showed that the field application of the recommended and double the recommended dose of PYR resulted in significant impairment of soil microbial diversity and functioning.

Conversely, the dissipation of PYR, regardless of microplastics, was strongly delayed in the presence of ABZ in Greek soil, as reflected in its significantly higher DT_50_ values in the ABZ-treated samples and in French soil, as implied by the significantly higher residual levels of PYR in the ABZ-treated samples. Lagos et al.^46^ showed that ABZ at concentrations equal to the ones used in our study imposed strong inhibitory effects on the diversity and abundance of most microbial groups in two tested soils, including bacteria and fungi that are known to be the key drivers in the degradation of xenobiotics in soil. We posit that ABZ had a strong and broad inhibitory effect on the soil microbiota, an effect previously reported for other compounds,^89,90^ with reciprocal deceleration of the soil dissipation of PYR which is primarily a microbially mediated process.^91^

Besides bipartite interactions, we identified clear tripartite interactions between microplastics ABZ and PYR with strong signatures on the dissipation of ABZ and PYR. Regarding ABZ, several lines of evidence suggest the involvement of tripartite interactions in its dissipation like (a) the negation of the stimulatory effect of microplastics on the dissipation of ABZ in Greek soil in the presence of PYR, (b) the further deceleration in the dissipation of the total residues of ABZ in the microplastic-amended samples in Greek soil in the presence of PYR (compared to PYR-untreated samples), and (c) the delayed dissipation of ABZ in French soil in the presence of both PYR and microplastics. These results are most probably associated with the broad inhibitory effects of PYR on the soil microbiota^52,88^ and on the plastisphere microbiota where PYR is preferentially adsorbed,^85^ which mediate ABZ. Regarding PYR, the inhibitory effect of ABZ on its dissipation in Greek soil was further exacerbated in the presence of microplastics. We suggest that microplastics further reduce the bioavailability of PYR in soil, based on its well-documented high adsorption affinity on plastic surfaces,^77,80^ prolonging its soil persistence.

Overall, the interaction of microplastics with other organic pollutants and the soil microbiota could lead to reciprocal effects on the processes that determine the environmental fate of the pollutants. These interactions are complex and often varied across soils although direct comparisons between soils and further determination of the parameters driving these soil-dependent responses were beyond the scope of the study. We provide first evidence for the effects of microplastics on the dissipation of the anthelminthic ABZ and the fungicide PYR. Microplastics showed distinct effects on the dissipation of ABZ and PYR, which did not vary across microplastic types and concentrations; they accelerated ABZ dissipation in Greek soil but had no effect on the dissipation of PYR. Besides microplastics, we observed strong bipartite interactions between ABZ and PYR, where ABZ decelerated the dissipation of PYR in two of the soils tested. Interestingly, this adverse effect of ABZ on the soil dissipation of PYR was more prominent in the presence of microplastics, offering a clear example of tripartite interactions between PYR, ABZ, and microplastics. These complex bipartite and tripartite interactions between microplastics–pesticides–anthelminthics, as identified in our study, strongly influence the dissipation of pollutants in agricultural soils with serious implications for environmental quality and soil health, yet they have not been adequately considered in current regulatory frameworks.

## Supplementary Material



## Notes

Graphs of the fitted kinetic models corresponding to dissipation data from the different treatments (PDF)

## ⊥

University of Maryland, Department of Geology, College Park, MD 20742, United States
