# Supplementary material for: Pesticide, Veterinary Medicines, and Microplastics: Bipartite and Tripartite Interactions Drive the Transformation of Albendazole and Pyraclostrobin in Agricultural Soils
Source: J Agric Food Chem. 2025 Dec 18;73(52):33005–18. doi: 10.1021/acs.jafc.5c09441 (PMC12766733; doi:10.1021/acs.jafc.5c09441)
Supplement: Supplementary file 1 [file jf5c09441_si_001.pdf]

Pesticide, veterinary medicines and microplastics: bipartite and tripartite interactions drive the transformation of albendazole and pyraclostrobin in agricultural soils

Eleni R. Lamprou[1], Hongfei Liu[1,4], Myriel Cooper[2], Stathis Lagos[1], Joana MacLean[3], Clemence Thiour-Mauprivez[2], Aymé Spor[2], Fabrice Martin-Laurent[2], Matthias C. Rillig[3], Dimitrios G. Karpouzas[1 *]

[1] University of Thessaly, Department of Biochemistry and Biotechnology, Laboratory of Plant and Environmental Biotechnology, Larissa 41500, Viopolis, Greece

[2] INRAE, Agroecologie - 17 Rue Sully, 21000, Dijon, France Univ. Bourgogne Europe Institut Agro, INRAE, Agroécologie, Dijon, France

[3] Freie Universität Berlin, Institute of Biology, Altensteinstraße 6, 14195 Berlin, Germany

[4] Current address: University of Maryland, Department of Geology, College Park, MD 20742, United States

[*]Corresponding author

Dimitrios G. Karpouzas

Tel. +302410565294

Email. dkarpouzas@uth.gr,

Supporting information-Kinetic models

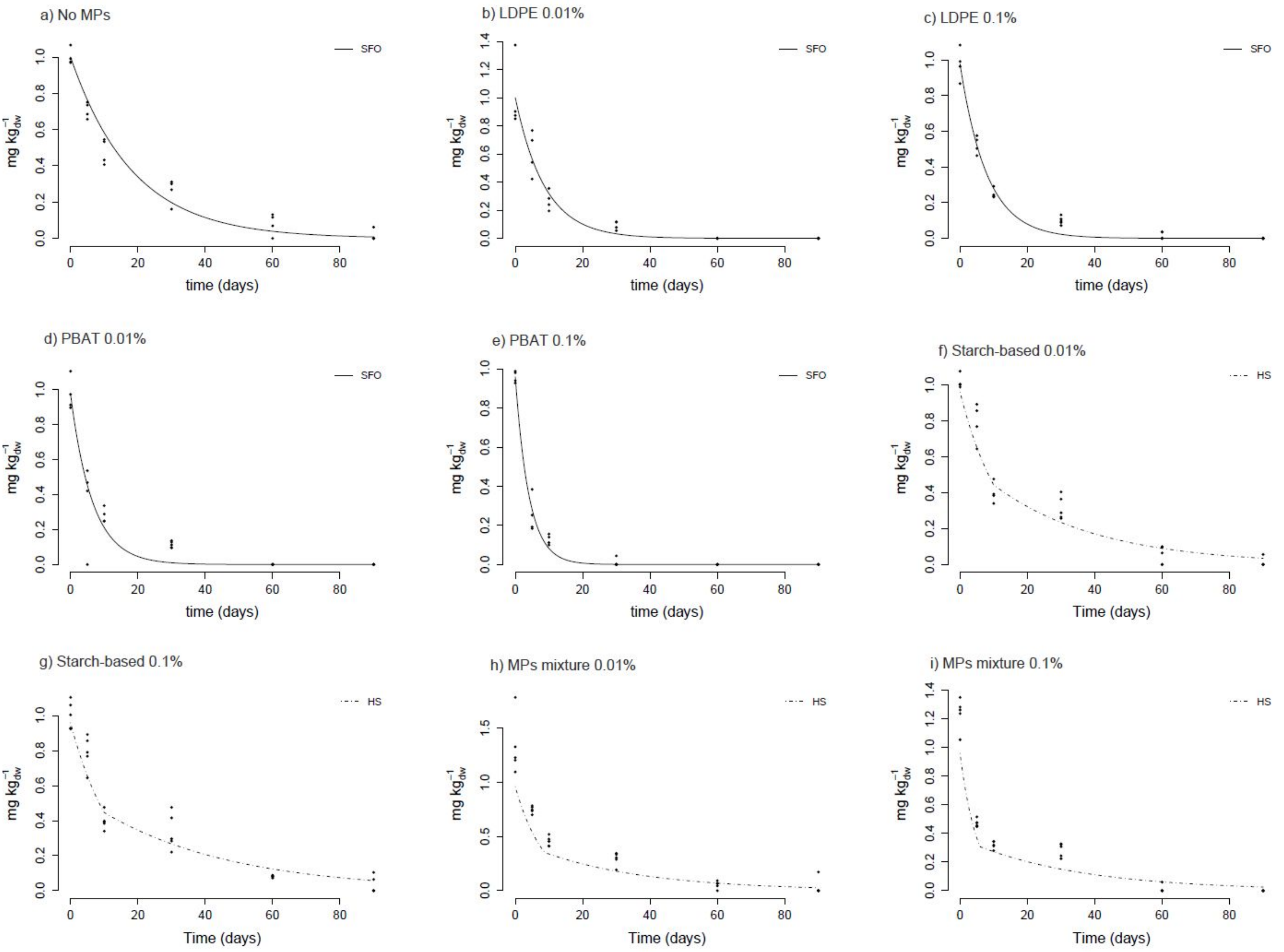

Figure S1. The dissipation patterns of albendazole (ABZ) in Greek soil in the presence of different types of microplastics (MPs) applied at two concentrations (0.01% and 0.1%). The Single-First-Order (SFO) or the Hockey-Stick (HS) kinetic models were fitted to the dissipation data in the different treatments : (a) No MPs, (b) LDPE 0.01%, (c) LDPE 0.1%, (d) PBAT 0.01%, (e) PBAT 0.1%, (f) Starch-based 0.01%, (g) Starch-based 0.1%, (h) MPs Mixture 0.01%, (i) MPs mixture 0.1%.

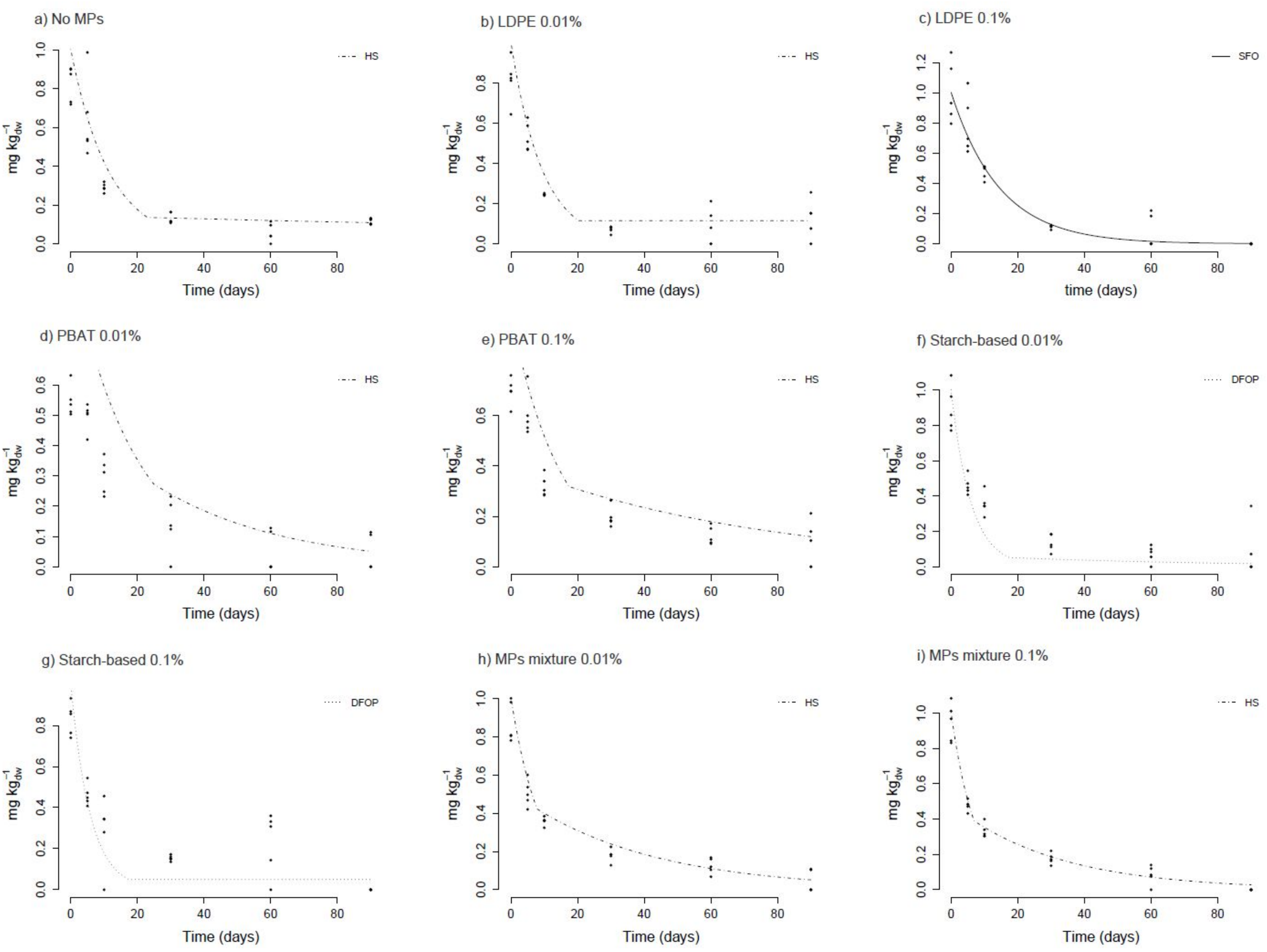

Figure S2. The dissipation patterns of albendazole (ABZ) in Greek soil in the presence of different types of microplastics (MPs), applied at two concentrations (0.01% and 0.1%), and pyraclostrobin. The Double First-Order in parallel (DFOP), and the Hockey-Stick (HS) kinetic models were fitted to the dissipation data in the different treatments : (a) No MPs, (b) LDPE 0.01%, (c) LDPE 0.1%, (d) PBAT 0.01%, (e) PBAT 0.1%, (f) Starch-based 0.01%, (g) Starch-based 0.1%, (h) MPs Mixture 0.01%, (i) MPs mixture 0.1%.

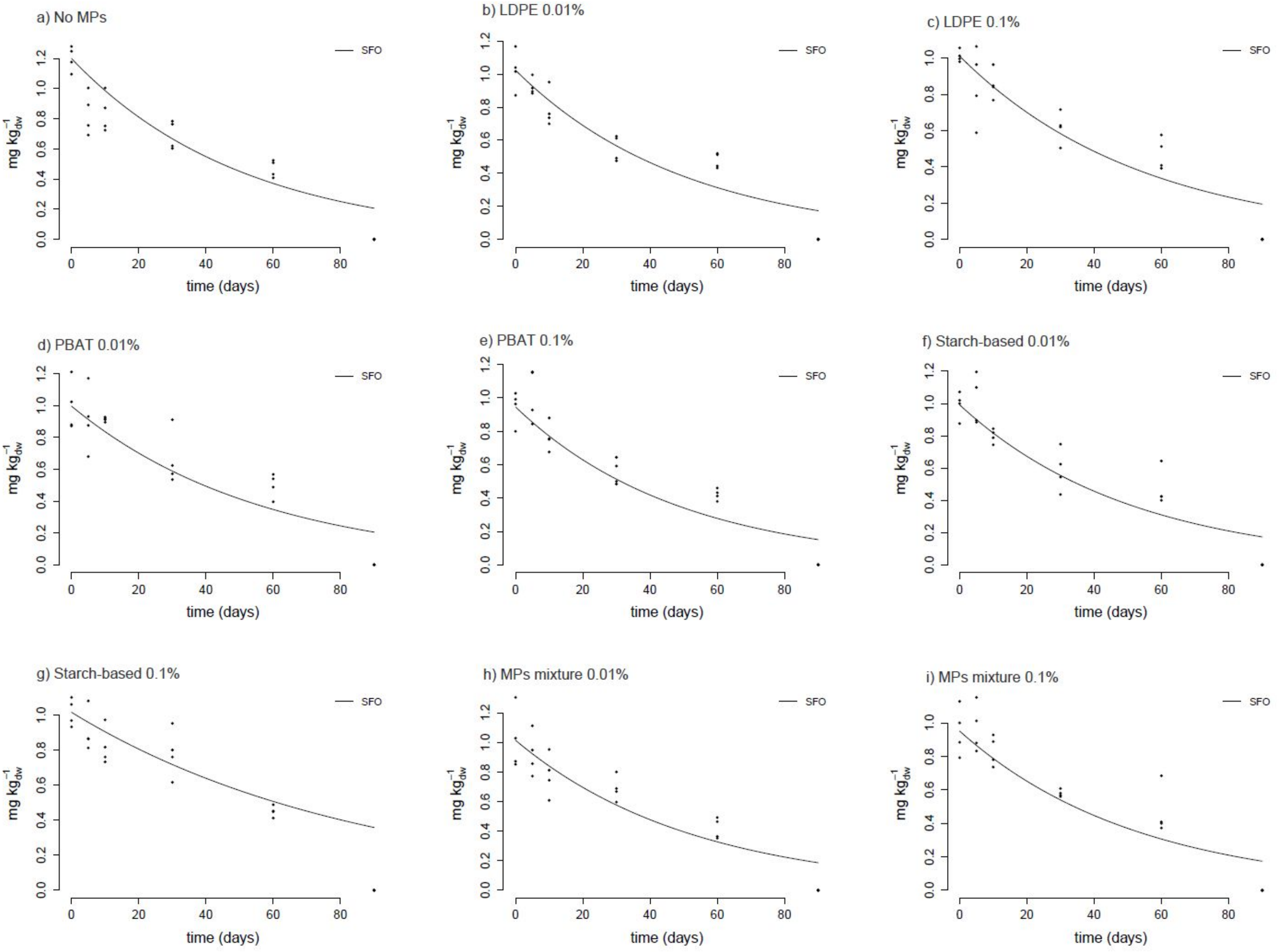

Figure S3. The dissipation patterns of pyraclostrobin (PYR) in Greek soil in the presence of different types of microplastics (MPs) applied at two concentrations (0.01% and 0.1%). The Single First-Order (SFO) kinetic model was fitted to the dissipation data in the different treatments: (a) No MPs, (b) LDPE 0.01%, (c) LDPE 0.1%, (d) PBAT 0.01%, (e) PBAT 0.1%, (f) Starch-based 0.01%, (g) Starch-based 0.1%, (h) MPs Mixture 0.01%, (i) MPs mixture 0.1%.

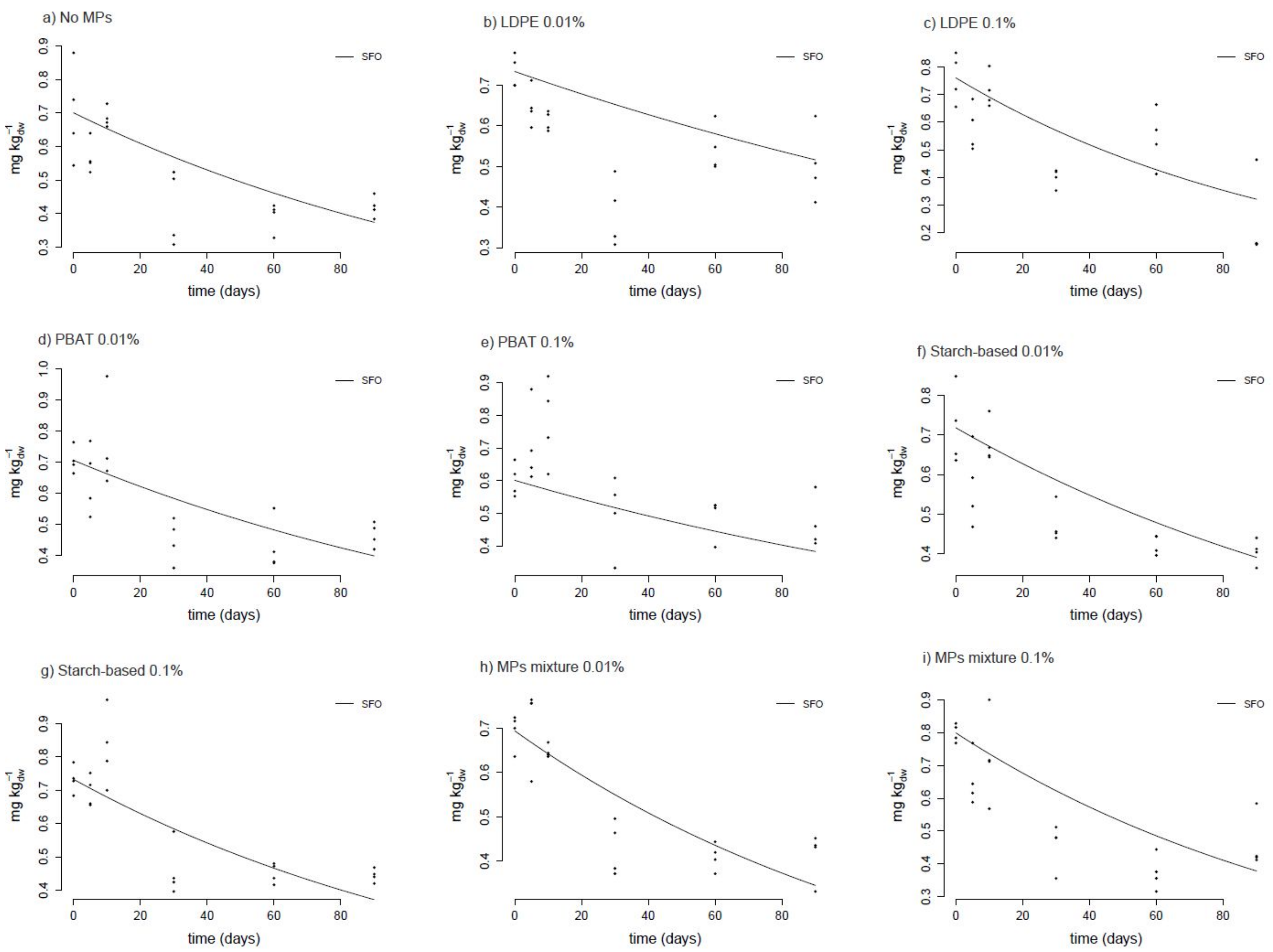

Figure S4. The dissipation patterns of pyraclostrobin (PYR) in Greek soil in the presence of different types of microplastics (MPs), applied at two concentrations (0.01% and 0.1%), and albendazole (ABZ). The Single First-Order (SFO) kinetic model was fitted to the dissipation data in the different treatments: (a) No MPs, (b) LDPE 0.01%, (c) LDPE 0.1%, (d) PBAT 0.01%, (e) PBAT 0.1%, (f) Starch-based 0.01%, (g) Starch-based 0.1%, (h) MPs Mixture 0.01%, (i) MPs mixture 0.1%.
